# Proteomics of the wheat tan spot pathogen *Pyrenophora tritici*-*repentis*

**DOI:** 10.1186/s13104-018-3936-y

**Published:** 2018-11-29

**Authors:** Caroline S. Moffat, Thomas Stoll, Paula Moolhuijzen

**Affiliations:** 1 Centre for Crop and Disease Management, School of Molecular and Life Sciences, Curtin University, Perth, WA Australia; 2 Protein Discovery Centre, QIMR Berghofer Medical Research Institute, 300 Herston Rd, Herston, QLD Australia

**Keywords:** Tan spot, Yellow spot, *Pyrenophora tritici*-*repentis*, Wheat, Necrotroph, Fungus, Pathogen, Proteome

## Abstract

**Objectives:**

The fungus *Pyrenophora tritici*-*repentis* is a major pathogen of wheat worldwide, causing the leaf spotting disease tan spot. To best inform approaches for plant genetic resistance, an understanding of the biology and pathogenicity mechanisms of this fungal pathogen is essential. Here, intracellular and extracellular proteins of *P. tritici*-*repentis* were extracted, and peptides analysed via high-resolution mass spectrometry. Our objective was to generate a useful proteomics resource for *P. tritici*-*repentis*. A survey of proteins secreted by the pathogen into culture filtrate is especially useful, as these are likely to come in direct contact with the wheat host and may play important roles in infection/pathogenicity. The peptide data presented herein, has also been used to successfully verify and refine in silico predicted *P. tritici*-*repentis* gene annotations, through the validation of alternative splicing and reading frame shifts.

**Data description:**

The data sets presented consist of peptide spectra of the extracellular and intracellular proteomes of three *P. tritici*-*repentis* isolates. Peptide matches to translated transcripts of the North American reference isolate Pt-1C-BFP are also provided.

## Objective

*Pyrenophora tritici*-*repentis* is a necrotrophic fungal pathogen and is the causal agent of tan spot, an economically significant global disease of wheat, typified by leaf lesions and reduced grain yield. Understanding the biology and pathogenicity mechanisms of this fungus is important for informing breeding approaches to host genetic resistance. The data described in this article is derived from mass spectrometry based proteomics, to identify fungal proteins produced under vegetative growth conditions.

Both intracellular and extracellular (culture filtrate) proteins were extracted from three isolates of *P. tritici*-*repentis*: two race 1 Australian isolates (PtrM4 and Ptr11137) and the race 5 North American isolate PtrDW5. Proteomes were analysed by protein OFFGEL fractionation followed by LC–MS/MS.

Our objective was to generate a useful and accessible *P. tritici*-*repentis* proteomics data resource. The data presented here is, to our knowledge, the first report of detectable *P. tritici*-*repentis* proteins in these three isolates. A previous proteomics study compared the secretomes and mycelial proteomes of an Algerian race 5 isolate and an avirulent Canadian race 4 isolate, however, only proteins detected as differentially abundant between the two isolates were subjected to MS/MS [1].

Since extracellular proteins typically include secreted cell wall degrading enzymes and necrotrophic effectors, this data is useful for identifying potential pathogenicity-related proteins, while the intracellular proteins may be helpful for identifying proteins involved in cell maintenance and growth. Furthermore, the peptide data can be used to validate and refine gene annotations, by identifying and resolving any peptide/annotation conflicts, intron/exon boundaries and identifying new loci [2]. This is especially useful for annotating genes that are complicated due to abnormal G:C content, coding frame shifts, rare intron donor and acceptor sites and unusual exon lengths.

## Data description

Here, we present six datasets representing the intracellular and extracellular proteomes of three isolates of *P. tritici*-*repentis* (race 1: Ptr11137 and PtrM4; race 5: PtrDW5). Each dataset is comprised of a MS/MS spectra file (mgf) and an identification file (mzid), as shown in Table 1. The mzid files consist of peptides mapped to translated transcripts of the North American race 1 reference isolate Pt-1C-BFP. Data files have been deposited with the ProteomeXchange resource MassIVE [3].

**Table 1:** Overview of data files/data sets

Label	Name of data file/data set	File types (file extension)	Data repository and identifier (DOI or accession number)
Data set 1	PtrM4 extracellular spectra	Peak file (mgf) Result file for peptide hits to Pt-1C-BFP (mzid)	MassIVE (MSV000083060) ftp://massive.ucsd.edu/MSV000083060
Data set 2	PtrDW5 extracellular spectra	Peak file (mgf) Result file for peptide hits to Pt-1C-BFP (mzid)	MassIVE (MSV000083059) ftp://massive.ucsd.edu/MSV000083059
Data set 3	Ptr11137 extracellular spectra	Peak file (mgf) Result file for peptide hits to Pt-1C-BFP (mzid)	MassIVE (MSV000083061) ftp://massive.ucsd.edu/MSV000083061
Data set 4	PtrM4 intracellular spectra	Peak file (mgf) Result file for peptide hits to Pt-1C-BFP (mzid)	MassIVE (MSV000083062) ftp://massive.ucsd.edu/MSV000083062
Data set 5	PtrDW5 intracellular spectra	Peak file (mgf) Result file for peptide hits to Pt-1C-BFP (mzid)	MassIVE (MSV000083063) ftp://massive.ucsd.edu/MSV000083063
Data set 6	Ptr11137 intracellular spectra	Peak file (mgf) Result file for peptide hits to Pt-1C-BFP (mzid)	MassIVE (MSV000083064) ftp://massive.ucsd.edu/MSV000083064

As an example of data usefulness, the spectral data was utilized previously for direct-to-genome mapping of peptide sequences to support and validate unusual and novel gene annotations for all three isolates [2]. This included peptide alignment to coding sequences, putative untranslated regions (UTRs) and intronic regions to confirm codon translational frame shifts, as well as detecting the presence of genes that had previously been missed by automated annotation. A total of 1568, 1868 and 1681 genes were supported for isolates PtrM4, Ptr11137 and PtrDW5 respectively, including 79, 78 and 97 predicted candidate effector genes [2].

### Methodology

#### Protein sample preparation

Extracellular proteins of the three isolates were obtained from 3-week old Fries 3 liquid culture filtrates that had been sequentially filtered [4] and extracted via TCA/acetone as described previously [2]. Briefly, culture filtrate was dialyzed, incubated with TCA/acetone solution in a 1:4 part ratio and centrifuged. Pellets were resuspended in 100% acetone, washed three times with acetone/Tris, air-dried and resuspended in 20 mM Tris pH 7. Any residual Tris was removed by dialysis.

Intracellular proteins of each isolate were obtained from mycelia of 3-day old minimal media liquid cultures. Mycelia were flash-frozen in liquid nitrogen, freeze-dried overnight and ground into a fine powder. Proteins were solubilized in 10 mM Tris–Cl pH 7 prior to centrifugation as previously described [2]. The supernatant was desalted and concentrated via TCA/acetone precipitation [2].

#### LC–MS/MS and data analysis

All protein samples were quantified via BCA and run on a 16.5% Tris/tricine gel to check for integrity. Samples were separated into 24 fractions by isoelectric focusing (OFFGEL) and the proteins of each fraction were then reduced, alkylated with iodoacetamide and trypsin-digested as described previously [5]. Peptides were separated on a C18 column via liquid chromatography and analysed by high-resolution mass spectrometry (LTQ Orbitrap Velos and XL) [2, 5].

A proteome search of the mass spectral data was conducted against the translated transcript database of the American reference isolate Pt-1C-BFP [6], as well as contaminant sequences of keratins, trypsin, BSA, plus a decoy database (reversed Pt-1C-BFP database) using the Mascot search engine [7] (variable modifications: Ox(M), Deamidation (N,Q); fixed modification: Carbamidomethyl (C); peptide tolerance.: 20 ppm, MSMS tolerance.: 0.8 Da; trypsin, 2 missed cleavages). Peptide/protein identifications were validated with Scaffold3 [8] with a minimum of 2 peptides, 95% peptide probability and 99% protein probability. The combined peak lists and peptide and protein identification results were exported using Scaffold4 with a 0.01 threshold FDR, and have been deposited with MassIVE [3] a full member of the ProteomeXchange consortium and are available for download.

## Limitations

The peptide hits are relative to the isolate Pt-1C-BFP, and therefore isolate-specific peptides may be missing. Peptides were searched against translations of predicted transcripts of Pt-1C-BFP, therefore will not match novel unannotated genes in Pt-1C-BFP. Only one biological replicate was utilized for each protein sample. Extracellular and intracellular peptides detected are constrained by the fungal growth conditions utilised and the time point that the tissue was harvested.

## Abbreviations

LC–MS/MS: liquid chromatography tandem–mass spectrometry
MS: mass spectrometry
TCA: trichloroacetic acid
UTR: untranslated region
CDS: coding sequence
BCA: bicinchoninic acid

## References

[1] Cao T, Kim YM, Kav NN, Strelkov SE (2009). A proteomic evaluation of *Pyrenophora tritici-repentis*, causal agent of tan spot of wheat, reveals major differences between virulent and avirulent isolates. Proteomics.

[2] Moolhuijzen P, See PT, Hane JK, Shi G, Liu Z, Oliver RP, Moffat CS (2018). Comparative genomics of the wheat fungal pathogen *Pyrenophora tritici-repentis* reveals chromosomal variations and genome plasticity. BMC Genomics.

[3] Goscinski WJ, McIntosh P, Felzmann UC, Maksimenko A, Hall CJ, Gureyev T, Thompson D, Janke A, Galloway G, Killeen NEB, Raniga P, Kaluza O, Ng A, Poudel G, Barnes D, Nguyen T, Bonnington P, Egan GF (2014). The Multi-modal Australian ScienceS Imaging and Visualisation Environment (MASSIVE) high performance computing infrastructure: applications in neuroscience and neuroinformatics research. Front Neuroinform.

[4] Moffat CS, See PT, Oliver RP (2014). Generation of a ToxA knockout strain of the wheat tan spot pathogen Pyrenophora tritici-repentis. Mol Plant Pathol.

[5] Anderson JP, Hane JK, Stoll T, Pain N, Hastie ML, Kaur P, Hoogland C, Gorman JJ, Singh KB (2016). Proteomic analysis of *Rhizoctonia solani* identifies infection-specific, redox associated proteins and insight into adaptation to different plant hosts. Mol Cell Proteomics.

[6] Manning VA, Pandelova I, Dhillon B, Wilhelm LJ, Goodwin SB, Berlin AM, Figueroa M, Freitag M, Hane JK, Henrissat B (2013). Comparative genomics of a plant-pathogenic fungus, *Pyrenophora tritici-repentis*, reveals transduplication and the impact of repeat elements on pathogenicity and population divergence. G3-genes. Genom Genet.

[7] Perkins DN, Pappin DJ, Creasy DM, Cottrell JS (1999). Probability-based protein identification by searching sequence databases using mass spectrometry data. Electrophoresis.

[8] Searle BC (2010). Scaffold: a bioinformatic tool for validating MS/MS-based proteomic studies. Proteomics.

